# Supplementary material for: Robust optimal design of FOPID controller for five bar linkage robot in a Cyber-Physical System: A new simulation-optimization approach
Source: PLoS One. 2020 Nov 30;15(11):e0242613. doi: 10.1371/journal.pone.0242613 (PMC7703880; doi:10.1371/journal.pone.0242613)
Supplement: Attachment — Submitted filename: Response to Reviewers--03 November 2020.docx [file pone.0242613.s003.docx]

The authors would cordially thank the respected associate editor and would appreciate esteemed reviewers for the useful comments and suggestions on the structure and contents of our manuscript. With respect to each comment, we have carefully modified the manuscript. The details of corrections are listed in the following point by point. The changes we made in the revised manuscript are marked in blue color. Politely, the manuscript has been resubmitted to the journal. We look forward to your positive response.

********************************************

**Reviewer #1:**

My comments have been addressed.

**Reviewer #2:**

1. Please label the lines in Figures 7 and 8.

**Response:** The authors would be thankful for your insightful comment. Both figures have been revised accordingly to include the labels.

2. The results need to be supported by the statistical analysis such as t-test.

**Response:** Thank you so much for your helpful comment. Two common statistical tests including the $t$-test and the Wilcoxon signed ranks test have been added in the new revised paper to support obtained results concerning the pairwise comparison of the proposed algorithm with three stochastic optimizers including PSO, GWO, and ALO. The statistical test results and the relevant discussion are provided in Table 8 and Section 4.5.1.
